# Targeting the PI3K and MAPK pathways to improve response to HER2-targeted therapies in HER2-positive gastric cancer

**DOI:** 10.1186/s12967-021-02842-1

**Published:** 2021-05-01

**Authors:** M. Janusz Mezynski, Angela M. Farrelly, Mattia Cremona, Aoife Carr, Clare Morgan, Julie Workman, Paul Armstrong, Jennifer McAuley, Stephen Madden, Joanna Fay, Katherine M. Sheehan, Elaine W. Kay, Ciara Holohan, Yasir Elamin, Shereen Rafee, Patrick G. Morris, Oscar Breathnach, Liam Grogan, Bryan T. Hennessy, Sinead Toomey

**Affiliations:** 1 Medical Oncology Group, Department of Molecular Medicine, Royal College of Surgeons in Ireland, RCSI Smurfit Building, Beaumont Hospital, Dublin 9, Ireland; 2 Data Science Centre, Royal College of Surgeons in Ireland, Dublin, Ireland; 3 Department of Histopathology, Royal College of Surgeons in Ireland, Dublin, Ireland; 4 Department of Medical Oncology, St. James’s Hospital, Dublin, Ireland; 5 Department of Medical Oncology, Beaumont Hospital, Dublin, Ireland

**Keywords:** HER2-positive gastric cancer, Signalling pathway activation, PI3K, MAPK, Targeted therapies, Somatic mutations, Treatment resistance

## Abstract

**Background:**

Aberrant PI3K signalling is implicated in trastuzumab resistance in HER2-positive gastric cancer (GC). The role of PI3K or MEK inhibitors in sensitising HER2-positive GCs to trastuzumab or in overcoming trastuzumab resistance is unclear.

**Methods:**

Using mass spectrometry-based genotyping we analysed 105 hotspot, non-synonymous somatic mutations in *PIK3CA* and ERBB-family (*EGFR*, *ERBB2*, *ERBB3* and *ERBB4*) genes in gastric tumour samples from 69 patients. A panel of gastric cell lines (N87, OE19, ESO26, SNU16, KATOIII) were profiled for anti-proliferative response to the PI3K inhibitor copanlisib and the MEK1/2 inhibitor refametinib alone and in combination with anti-HER2 therapies.

**Results:**

Patients with HER2-positive GC had significantly poorer overall survival compared to HER2-negative patients (15.9 months vs. 35.7 months). Mutations in *PIK3CA* were only identified in HER2-negative tumours, while ERBB-family mutations were identified in HER2-positive and HER2-negative tumours. Copanlisib had anti-proliferative effects in 4/5 cell lines, with IC50s ranging from 23.4 (N87) to 93.8 nM (SNU16). All HER2-positive cell lines except SNU16 were sensitive to lapatinib (IC50s 0.04 µM–1.5 µM). OE19 cells were resistant to trastuzumab. The combination of lapatinib and copanlisib was synergistic in ESO-26 and OE-19 cells (ED50: 0.83 ± 0.19 and 0.88 ± 0.13, respectively) and additive in NCI-N87 cells (ED50:1.01 ± 0.55). The combination of copanlisib and trastuzumab significantly improved growth inhibition compared to either therapy alone in NCI-N87, ESO26 and OE19 cells (p < 0.05).

**Conclusions:**

PI3K or MEK inhibition alone or in combination with anti-HER2 therapy may represent an improved treatment strategy for some patients with HER2-positive GC, and warrants further investigation in a clinical trial setting.

**Supplementary Information:**

The online version contains supplementary material available at 10.1186/s12967-021-02842-1.

## Background

Gastric cancer (GC) is one of the most common cancers with over 1 million new cases each year, and despite a decrease in specific mortality, remains the second leading cause of cancer related death worldwide with over 780,000 deaths from the disease occurring annually [1]. The majority of patients diagnosed with GC present with advanced, incurable disease. Therapeutic advances in the treatment of the disease have been slow, and systemic chemotherapy remains the standard of care for treatment of advanced disease [2]. 5-year overall survival (OS) ranges from 57 to 79% in stage I, and 4% in stage IV [3]. Although resectable disease can potentially be cured by surgery with or without chemotherapy, metastatic recurrence rates remain high [4]. In metastatic disease, median survival is approximately 9 months in patients treated with chemotherapy compared with 5 months for patients treated with supportive care alone [5].

Approximately 6–30% of GCs overexpress human epidermal growth factor receptor 2 (HER2), and expression is dependent on the location of the primary tumour [6–8]. HER2 plays a role in regulating cell survival and growth by acting through the PI3K/AKT/mTOR and RAS-MAPK pathways [9]. Although HER2-positivity correlates with pathological features of poor prognosi [10], the role of HER2-overexpression as a prognostic biomarker in GC is less well defined. Some studies have shown HER2-positivity to be a poor prognostic marker associated with worse survival [11, 12], while others have found no association between HER2-positivity and prognosis in both early and advanced stage disease [13, 14]. The phase III ToGA (Trastuzumab for Gastric Cancer) trial investigated the role of trastuzumab therapy in patients with HER2-positive advanced gastric or gastro-oesophageal junction cancer. Patients were randomized to receive either trastuzumab in combination with chemotherapy or chemotherapy alone. The addition of trastuzumab led to an increase in overall survival by 2.7 months compared to chemotherapy alone [8]. This led to the approval of trastuzumab as the first molecular targeted therapy for GC, however not all patients with HER2-positive GC respond to trastuzumab, and the majority of patients who respond develop resistance after a relatively short period of time [15]. Subsequent clinical trials with another anti-HER2 treatment, lapatinib, failed to show any survival advantage [16]. The identification of mechanisms underlying treatment resistance could potentially identify novel treatment options to improve trastuzumab sensitivity in HER2-positive GC.

Intracellular pathways, including the PI3K/AKT and RAS/RAF/MEK pathways are activated by ligand binding to ERBB-family members [17]. Anti-HER2 therapies block this signalling, either by binding ERBB2 at the cell surface or by directly inhibiting the kinase activity of both EGFR and ERBB2 [17]. Possible resistance mechanisms include constitutive activation of the PI3K/AKT pathway through somatic mutations in the PI3K pathway [18, 19]. Mutations in *PIK3CA,* which encodes the p110a subunit of PI3K, occur in approximately 20% of GCs [20], however *PIK3CA* mutations are less frequent in HER2-positive GC, occurring in only 2% of tumours [21].

Mutations in *EGFR*, *ERBB2*, *ERBB3* and *ERBB4* (known as ERBB-family mutations herein) occur in 12% of GCs (n = 434) [22, 23]. As ERBB-mediated effects are dependent on PI3K/AKT signalling, and ERBB-family mutations can activate the PI3K/AKT pathway it is likely that they have similar canonical signalling effects to PI3K pathway mutations. Because these mutations may play a role in the development of resistance to trastuzumab, our study aimed to determine the frequency of *PIK3CA* and/or ERBB-family mutations in gastric adenocarcinoma. We also investigated whether targeting the PI3K or MAPK pathway in vitro using copanlisib, a p110α/δ inhibitor, or refametinib, a MEK1/2 inhibitor, could potentially improve responses to anti-HER therapies in HER2-positive GCs and provide evidence to support a future Phase Ib clinical trial. These inhibitors were chosen as we have previously shown that copanlisib and refametinib can restore sensitivity to anti-HER2 therapies in vitro in HER2-positive breast cancer cell lines with acquired resistance to trastuzaumab and lapatinib, therefore we hypothesised that we would observe a similar effect in HER2-positive GC cell lines [24, 25].

## Methods

### Patients and samples

Archival formalin fixed paraffin embedded (FFPE) tumour samples were obtained from tumour banks under the auspices of Institutional Review Board approved protocols. Tumour selection was based on a diagnosis of GC, the availability of sufficient tissue from the tumour, and the availability of relevant clinical data. All patients were diagnosed between January 2006 and December 2012. Samples were assessed for HER2-positivity using immunohistochemistry (IHC) (Hercept Test, Dako, Denmark or PATHWAY anti-HER2 (4B5), Ventana, Tuscon, AZ, USA). Samples with an IHC score of 2 + were assessed subsequently with fluorescence in situ hybridisation (FISH). Clinical characteristics of the patients included in the study are described in Table 1.

### Sample preparation of FFPE tumour tissue and MassArray genotyping

From each tumour sample 6 × 10 µM sections were cut from the paraffin block. Hematoxylin and eosin staining was performed on the first and last sections and checked by an experienced Pathologist to ensure that there was tumour present. If the tumour content was lower than 30%, tumour area was macrodissected. DNA was extracted using an All Prep DNA FFPE kit (Qiagen), as per manufacturer’s instructions. DNA concentration was calculated using the Qubit dsDNA kit.

Mass Spectrometry single nucleotide polymorphism genotyping technology (Agena Biosciences, Hamburg, Germany) was applied to DNA extracted from the FFPE samples to detect a total of 108 nonsynonymous somatic mutations in *PIK3CA, EGFR, ERBB2, ERBB3 and ERBB4*. Mutations in ERBB-family genes were identified using publically available data from the TCGA database and a literature search [23]. AVSIFT and Mutation Assessor scores were used to determine the ERBB-family mutations that were likely to be deleterious, and result in activation of the PI3K and MAPK signalling pathways. The full list of mutations is listed in the supplementary data (Additional File 1: Table S1). Matrix chips were analysed on an Agena MassArray MALDI-TOF system. Visual inspection and Typer Software were used to identify genotypes based on mass spectra. Reactions where > 15% of the resultant mutant mass ran in the mutant site were scored as positive.

### Protein extraction and RPPA analysis of HER2-positive gastric tumours and cell lines

From each tumour 5 × 10 µM sections were cut from the paraffin block. Protein extraction was carried out as previously described [26] using a 20-mM Tris buffer pH 9 containing 2% SDS and protease inhibitors (Roche Applied Science Cat. #04693116001 and 04906845001) lysis buffer.

Reverse phase protein array (RPPA) analysis was carried out as previously described [24, 27]. The full list of antibodies used are in supplementary data (Additional File 1: Table S2). The data was normalised by protein loading using the entire antibody panel. RPPA data is shown in Additional File 2.

### Cell culture

The GC cell lines NCI-N81, SNU16 and KATOIII were obtained from ATCC and ESO26 and OE19 were obtained from ECACC. All cell lines were cultured in RPM1-1640 media (Sigma) supplemented with 10% FBS and 1% Penicillin/Streptomycin, and maintained at 37 °C with 5% CO_2_. Cell line identity was confirmed by DNA fingerprinting (Source Biosciences). Cell lines were mycoplasma tested before and after in vitro experiments. Trastuzumab (21 mg/ml) was obtained from Beaumont Hospital pharmacy and was prepared in sterile water. Lapatinib (10.8 mM) was purchased from Selleckchem and stock solutions were prepared in dimethylsulfoxide (DMSO). The PI3K inhibitor copanlisib and the MEK1/2 inhibitor refametinib were obtained from Selleckchem and stocks (5 mM copanlisib; 10 mM refametinib) were prepared in 100% DMSO with 10 mM TFA and 100% DMSO, respectively.

### HER2 immunohistochemistry

Cells were made into cell blocks using 1% low melting point agar before formalin-fixation and paraffin embedding. 3 μM sections for Her2 (4B5) IHC (Ventana, Cat. No 790-2991) were stained using the Ventana BenchMark XT stainer.

### Protein extraction from cell lines and western blotting

4 × 10^5^ cells were seeded in 6-well plates and grown until confluent. Total protein was extracted using 100 µl lysis buffer (15% NACL 1 M, 1% Triton-X 100, 5% TRIS, 14% phosphatase inhibitors 7X, 65% dH_2_O) Protein was quantified by the bicinchoninic acid (BCA) assay and stored at – 80 °C until analysis.

30–40 μg protein was subjected to electrophoresis through 4–12% Bis–Tris precast gels (Invitrogen, Thermo Fisher Scientific, USA). After transfer, the membranes were incubated with corresponding primary antibodies at 4 °C overnight and secondary antibodies at room temperature for 1 h. Primary antibodies and dilutions were as follows: 1:1000 rabbit S6K1 (#2708, Cell Signalling Technologies (CST)); 1:1000 p-S6K1 (T389, T412) (ab60948, Abcam); 1:3000 Akt (#9272, CST); 1:1000 p-Akt (Thr308) (#4056, CST); 1:3000 MAPK Erk1/2 (#9102, CST); 1:3000 p-MAPK Erk1/2 (Thr202, Tyr204) (#9101, CST); 1:1000 PI3K p110α (#4255, CST). All membranes were additionally probed for ß-actin (1:3000, A5316, Sigma-Aldrich). Antibody binding was visualised by electrochemiluminescence reagents (Immobilon Western, Millipore Corporation, USA) for 5 min or 1 min for ß-actin and images were developed digitally (Amersham Imager 600, GE Healthcare).

### Proliferation assays

3 × 10^4^ cells were seeded in 96 well plates. Plates were incubated overnight at 37 °C to allow cells to adhere. Drugs were added to the plates and incubated at 37 °C. Copanlisib and refametinib were combined with lapatinib in cell lines at ratios determined from the IC50 values of the single agent drugs. Following 5-day incubation, all media was removed from the plates and the plates were washed once with PBS. Proliferation was measured in KATO III, ESO26, NCI-N87 and OE19 cells using the acid phosphatase assay, as previously described [28] and in SNU16 cells using the MTS assay. A minimum of triplicate biological assays were performed for each experiment (See Table 2).

**Table 1:** Clinical characteristics of the HER2-positive and HER2-negative patients

	HER2-positive (n = 29)	HER2-negative (n = 40)
Median age at diagnosis	64.5	78
	Range 32–79	Range 40–87
Gender		
Male	20 (69%)	22 (55%)
Female	9 (31%)	18 (45%)
Stage at diagnosis		
2	3 (10.3%)	2 (5%)
3	5 (17.2%)	9 (22.5)
4	18 (62.1%)	22 (55%)
Unknown	3 (10.3%)	7 (17.5%)
Treatment		
None	3 (10.3%)	28 (70%)
Trastuzumab-based	17 (58.6%)	0 (0%)
Chemotherapy alone	9 (31%)	12 (30%)
Tumour location		
Stomach	13 (44.8%)	21 (52.5%)
Gastro-oesophageal junction	16 (55.2%)	19 (47.5%)

In the HER2-positive patient cohort trastuzumab was given in combination with oxaliplatin and capecitabine or fluorouracil in 14 patients, and with fluorouracil alone in three patients. In the HER2-negative cohort, seven patients received fluorouracil and leucovorin, one patient received docetaxol and carboplatin, one patient received docetaxol, carboplatin and fluorouracil, one patient received oxaliplatin and fluorouracil and two patients received capecitabine alone

### Statistical analysis

Kaplan–Meier curves for overall survival (OS) and progression free survival (PFS) were statistically analyzed by the log rank test. Differences in mutation frequencies between HER2-positive and HER2-negative tumours and differences in protein expression between wildtype and mutated tumours were determined using students *t*-test. IC_50_ and combination index (CI) values at effective dose 50 (ED_50_) were calculated using CalcuSyn software (Biosoft). A CI value of < 0.9 is considered synergistic, 0.9–1.1 is considered additive and > 1.1 is considered antagonistic. A Kruskal–Wallis non-parametric test was performed to compare trastuzumab alone, copanlisib or refametinb alone or the combination of trastuzumab and copanlisib or trastuzumab and refametinib, p < 0.05 was considered statistically significant. Statistical analysis was carried out using GraphPad Prism.

## Results

### Patient characteristics

Sixty-nine GC patients were included. Forty (58%) patients had HER2-negative disease and 29 (42%) patients had HER2-positive disease. Of the HER2-positive patients, 13 (44.8%) had gastric adenocarcinoma and 16 (55.2) had gastro-oesophageal junction adenocarcinoma. Of the HER2-negative patients, 21 (52.5%) had gastric adenocarcinoma and 19 (47.5%) had gastro-oesophageal junction adenocarcinoma. The median age at diagnosis was 64.5 years (range 32–79) in the HER2-positive cohort and 78 years (range 40–87) in the HER2-negative cohort. 20/29 (69%) and 22/40 (55%) of patients were male in the HER2-positive and HER2-negative cohorts, respectively. Stage at diagnosis was similar in both cohorts with the majority of patients diagnosed with stage 3 or 4 disease. Clinical characteristics are described in Table 1.

Of the HER2-positive patients 17/29 (58.6%) were treated with a trastuzumab containing regimen. Twelve patients (41.4%) did not receive trastuzumab as part of their treatment. Among the 17 patients who received trastuzumab, the drug was given in combination with oxaliplatin and capecitabine or fluorouracil in 14 patients, and with fluorouracil alone in three patients. In the HER2-negative patient cohort only 12/40 (30%) patients received systemic therapy. Seven patients received fluorouracil and leucovorin, one patient received docetaxol and carboplatin, one patient received docetaxol, carboplatin and fluorouracil, one patient received oxaliplatin and fluorouracil and two patients received capecitabine alone (Additional File 2: Table S3).

**Fig. 1:**
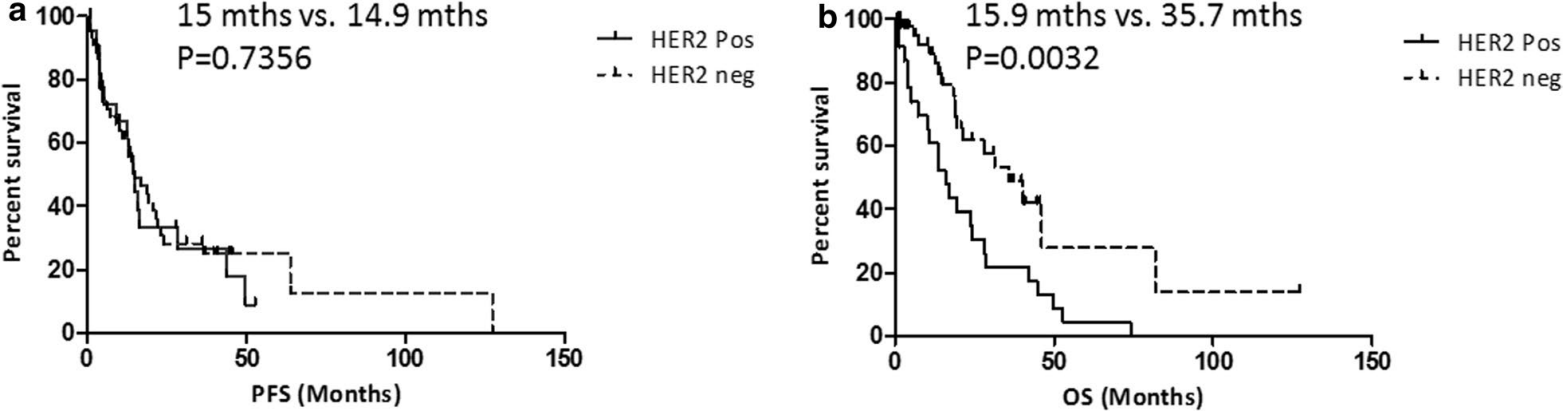
**a** Progression free survival (PFS) and **b** Overall Survival (OS) in HER2-positive (n = 29) and HER2-negative (n = 40) gastric cancer patients. p-values were calculated using the log rank (Mantel–Cox) test with GraphPad Prism

There was no difference in median PFS in patients with either HER2-positive or HER2-negative disease (15 months vs. 14.9 months; p = 0.7356), however median OS was significantly shorter in the HER2-positive patient cohort compared to the HER2-negative patient cohort (15.9 months vs. 35.7 months; p = 0.0032) (Fig. 1).

### Somatic mutation profiling of HER2-positive and HER2-negative gastric cancers

**Fig. 2:**
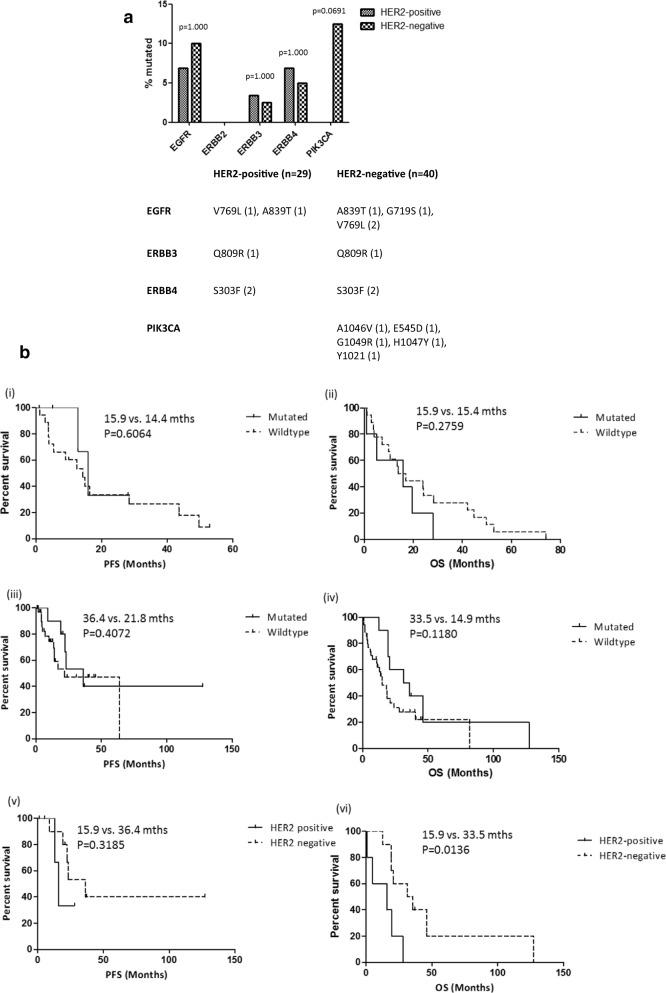
**a** Frequency of somatic PIK3CA and ERBB-family mutations in HER2-positive (n = 29) and HER2-negative (n = 40) tumour samples identified using the Agena MassArray system and **b** (i) Progression free survival (PFS) and (ii) overall survival (OS) in HER2-positive gastric cancer patients with and without PIK3CA and/or ERBB-family mutations; (iii) PFS and (iv) OS in HER2-negative gastric cancer patients with and without PIK3CA and/or ERBB-family gene mutations; (v) PFS and (vi) OS in HER2-positive and HER2-negative gastric cancer patients with PIK3CA and/or ERBB-family gene mutations. p-values were calculated using the log rank (Mantel–Cox) test with GraphPad Prism

*EGFR* mutations occurred more frequently in HER2-negative tumours than HER2-positive tumours, while mutations in other ERBB-family genes occurred more frequently in HER2-positive tumours than in HER2-negative tumours. *EGFR* mutations were identified in 4/40 (10%) HER2-negative tumours and 2/29 (6.9%) HER2-positive tumours. *ERBB3* mutations were identified in 1/40 (2.5%) HER2 negative tumours and 1/29 (3.4%) of HER2-positive tumours, while mutations in *ERBB4* were identified in 2/40 (5%) HER2-negative tumours and 2/29 (6.9%) HER2-positive tumours. *PIK3CA* mutations were identified in 5/40 (12.5%) HER2-negative tumours. No *PIK3CA* mutations were identified in HER2-positive tumours (Fig. 2a). No tumour samples had co-occurring *PIK3CA* and ERBB-family mutations.

There was no difference in median PFS or median OS in HER2-positive patients with and without *PIK3CA* or *ERBB*-family tumour mutations (Fig. 2b (i) and Fig. 2b (ii) or in HER2-negative patients with and without *PIK3CA* or ERBB-family tumour mutations (Fig. 2b (iii) and Fig. 2b (iv) There was no significant difference in median PFS between HER2-positive and HER2-negative patients with *PIK3CA* or ERBB-family mutations (Fig. 2b (v)), however HER2-positive patients with *PIK3CA* or ERBB-family mutations had significantly poorer median OS than HER2-negative patients with *PIK3CA* or ERBB-family mutations (15.9 months vs. 33.5 months; p = 0.0136) (Fig. 2b (vi)).

### Proteomic profile of HER2-positive gastric tumours

**Fig. 3:**
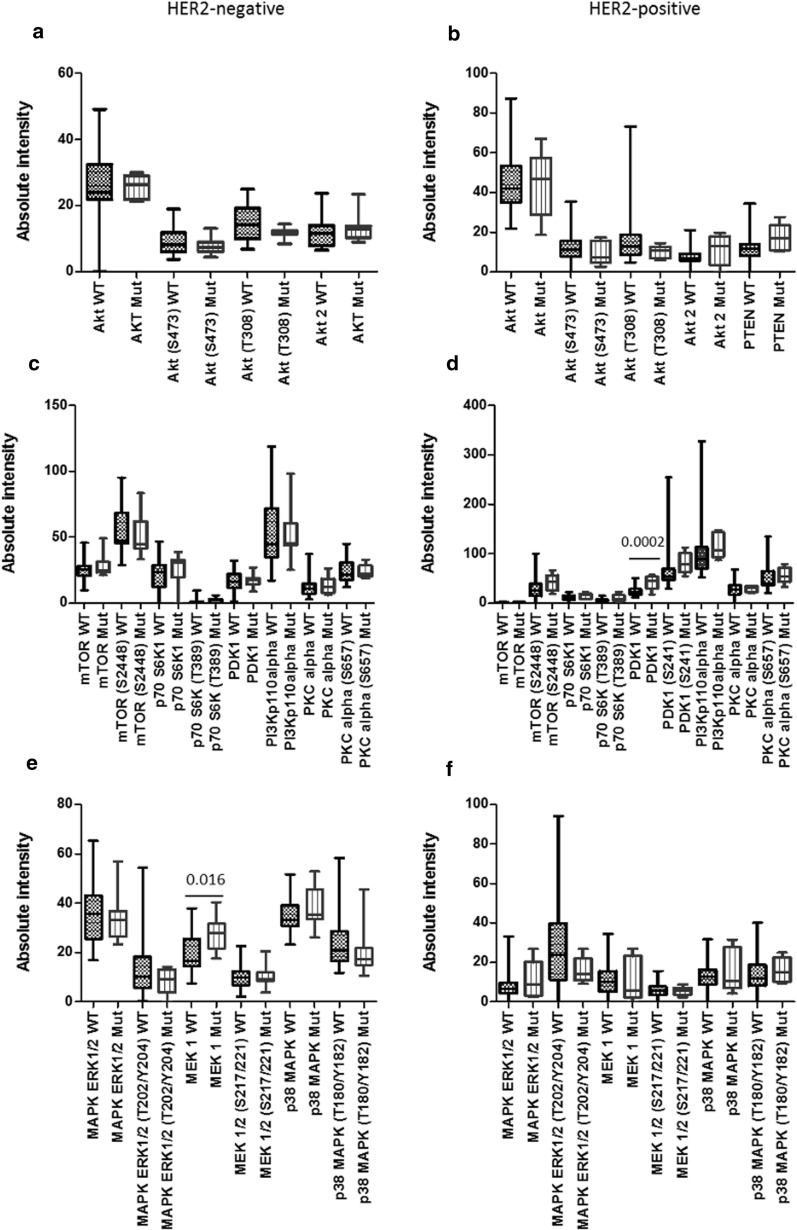
RPPA analysis displaying the levels of protein expression or phosphorylation of PI3K and MAPK signalling pathway proteins in the tumours of HER2-negative and HER2-positive patients with tumours harbouring PIK3CA and/or ERBB-family mutations. Differences in expression between wildtype and mutated tumours were determined using an unpaired *t*-test

Antibodies for RPPA analysis were chosen to represent multiple nodes on the PI3K/AKT and MAPK/ERK signalling pathways. Differential protein expression was examined between wildtype and PIK3CA and/or ERBB mutated tumours. There was no difference in AKT expression between wildtype and mutated tumours in either the HER2-negative or HER2-positive cohorts. PDK1 expression was significantly higher in the HER2-positive mutated tumours compared to the HER2-positive wildtype tumours (p = 0.0002). In the HER2-negative group expression of MEK1 was significantly higher in the mutated tumours compared to the wildtype tumours (p = 0.016) (Fig. 3).

### Baseline expression of HER2 and mutational status in a panel of established human gastric cancer cell lines

**Fig. 4:**
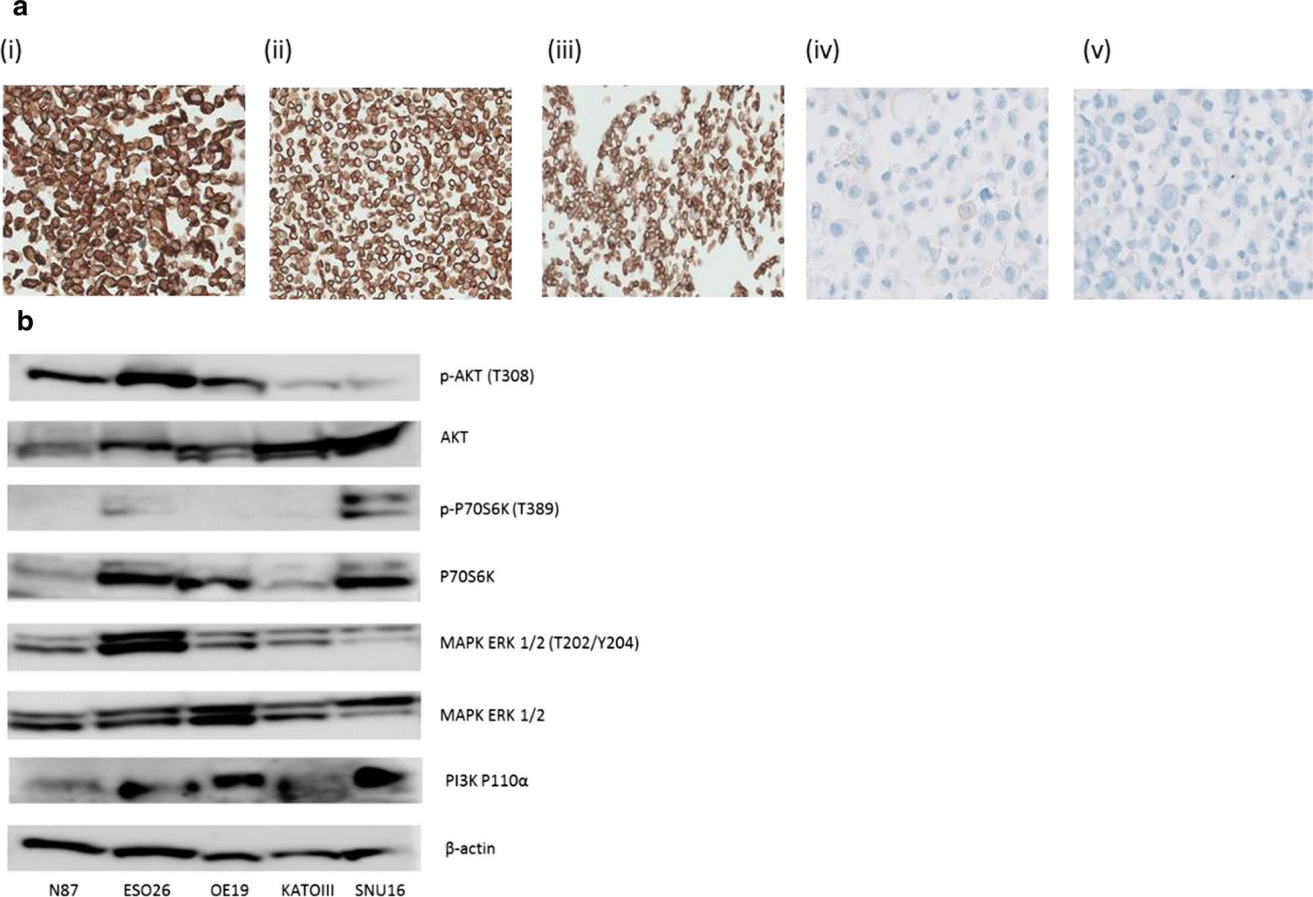
**a** HER2-staining by IHC in the gastric cancer cell lines (i) ESO26, (ii) NCI-N87, (iii) OE19, (iv) SNU16 and (v) KATOIII. Images i, ii and iii were taken at 10X magnification, while images iv, and v were taken at 20X magnification. **b** Western blot analysis of expression of p-AKT, AKT, p-P70S6K (T389), P70S6K, MAPK ERK 1/2 (T202/Y204), MAPK ERK 1/2, PI3K P110α, and β-actin in the gastric cancer cell lines NCI-N87, ESO26, OE19, KATOIII and SNU16. All gels were run under the same experimental conditions and the experiments were repeated three times. Representative images are shown

HER2 expression was assessed by immunohistochemistry in the human GC cell lines ESO26, NCI-N87, OE19, SNU16 and KATOIII (Fig. 4a). HER2 was highly expressed in ESO26, NCI-N87 and OE19 cells with a diffuse plasma membrane and cytosolic staining pattern. HER2 was weakly expressed (< 5% positive cells) in SNU16 and staining was restricted to the membrane. KATOIII cells did not express HER2.

We used publically available data from the cancer cell line encyclopaedia (CCLE) database to search for mutations in *EGFR, ERBB2, ERBB3, ERBB4, KRAS* and *BRAF* that might predispose to a different response to anti-HER2 therapies or PI3K or MAPK pathway inhibition. We confirmed the presence of mutations in these genes in the cell lines using the Agena MassArray system. ESO26 cells had co-occurring mutations in *ERBB4* (M772L) and *PIK3CA* (Q546H). NCI-N87 cells harboured two *ERBB2* mutations (F425L and L436V). SNU16 cells had a *KRAS* G12D mutation, while KATOIII cells had a *PIK3CA* L387L mutation. OE19 cells were wildtype for all mutations tested. No mutations in *EGFR, ERBB3* or *BRAF* were identified in any of the cell lines.

### Analysis of PI3K and MAPK pathway protein expression in gastric cancer cell lines

p-AKT expression was highest in the ESO26 cell line, which harbours a *PIK3CA* mutation. Expression of p-AKT was also high in the ERBB2 mutated NCI-N87 cell line and the wildtype OE19 cell line compared to the KATOIII and SNU-16 cell lines. p-P70S6K and PI3K P110α expression was highest in the *KRAS* mutated SNU16 cell line. ERK 1/2 (T202/Y204) expression was higher in the ERBB4/PIK3CA mutated ESO26 cell line than any of the other cell lines. Expression of all proteins was lowest in the wildtype HER2-negative KATOIII cell line (Fig. 4b).

### Single agent anti-HER2 therapy and PI3K and MAPK inhibitor sensitivity of gastric cancer cell lines

We investigated response to treatment with the anti-HER2 therapies trastuzumab and lapatinib in the cell lines. Lapatinib IC50s ranged from 39.8 ± 13.3 nM in NCI-N87 cells to 1660 ± 151.7 nM in OE19 cells. SNU16 and KATOIII cells, which have low and negative HER2 expression, respectively, are resistant to lapatinib (Table 2). Cell lines had varying sensitivity to single agent trastuzumab, achieving between 38.6 ± 2.1% growth inhibition in NCI-N87 cells to no growth inhibition in OE19 and KATOIII cells. The PI3K inhibitor copanlisib achieved an IC50 in all of the cell lines apart from OE19, ranging from 26.8 ± 9.7 nM in NCI-N87 cells to 102.6 ± 28.5 nM in SNU16 cells and was effective in cell lines regardless of their *PIK3CA* or ERBB-family mutation status. SNU16 cells, which have a *KRAS* G12D mutation, had a higher IC50 for copanlisib (102.6 ± 28.5 nM) than *KRAS* wildtype cell lines. We also investigated the effect of refametinib, a MEK1/2 inhibitor, in our panel of cell lines. IC50s for refametinib ranged from 2.75 ± 0.37 nM in OE19 cells to 6.05 ± 0.84 nM in KATOIII cells. ESO26 and NCI-N87 cells did not achieve an IC50 for refametinib at the concentrations of drug used in our study.

**Table 2:** Comparative mutational analysis as determined by Agena MassArray of mutations in EGFR, ERBB2, ERBB3, ERBB4, PIK3CA, KRAS and BRAF; IC_50_ values for copanlisib, refametinib, lapatinib, and the effect of trastuzumab on growth inhibition in a panel of gastric cancer cell lines

Cell line	HER2 status	Mutational Status							Response to targeted therapies			
		EGFR	ERBB2	ERBB3	ERBB4	PIK3CA	KRAS	BRAF	Copanlisib (nM)	Refametinib (µM)	Lapatinib (nM)	Trastuzumab % growth inhibition at 10 µg/ml
ESO26	Strongly positive	WT	WT	WT	M772L	Q546H	WT	WT	43.1 ± 9.6	> 10	290.8 ± 42.7	7.5 ± 5.7
NCI-N87	Strongly positive	WT	F425L L436V	WT	WT	WT	WT	WT	26.8 ± 9.7	> 10	39.8 ± 13.3	38.6 ± 2.1
OE19	Strongly positive	WT	WT	WT	WT	WT	WT	WT	> 1000	2.75 ± 0.37	1660 ± 151.7	− 1.8 ± 7.6
SNU16	Weakly positive	WT	WT	WT	WT	WT	G12D	WT	102.6 ± 28.5	5.6 ± 1.4	> 10,000	12 ± 3.1
KATOIII	Negative	WT	WT	WT	WT	L387L	WT	WT	65 ± 23.9	6.05 ± 0.84	> 10,000	-2.8 ± 7.4

Standard deviations are representative of triplicate experiments

### Combinations of lapatinib and copanlisib or refametinib are synergistic in some HER2-positive cancer cell lines

**Fig. 5:**
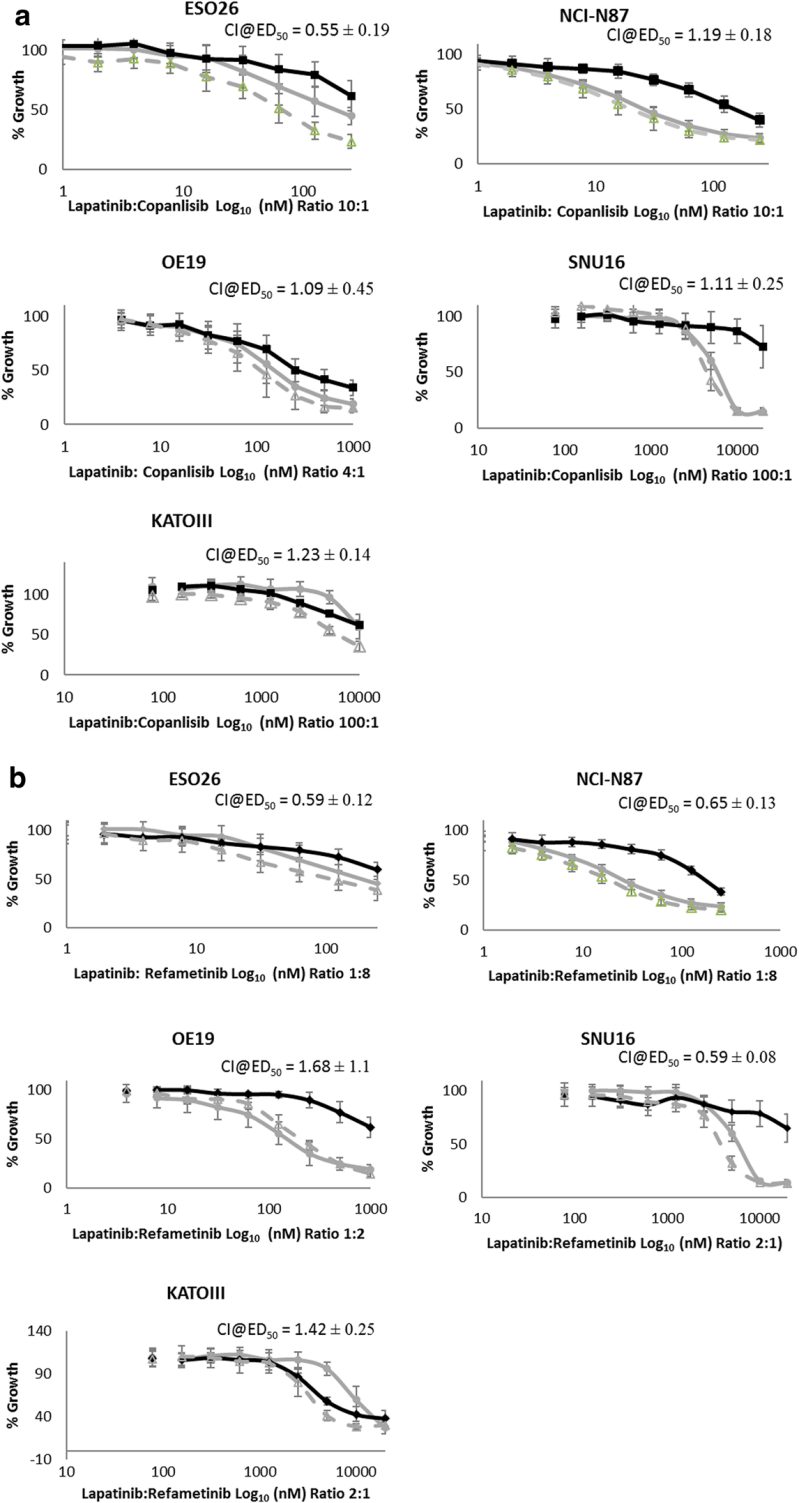

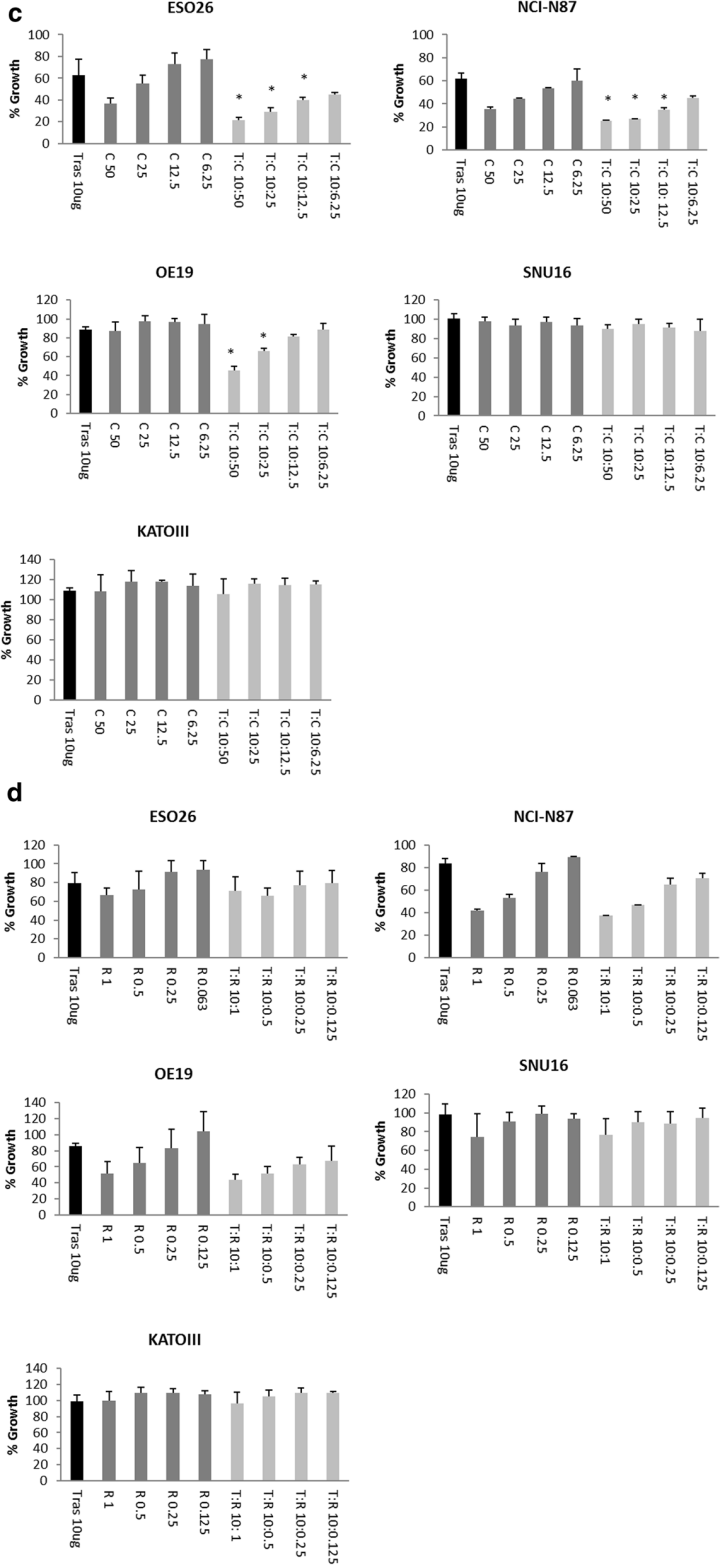
**a** Efficacy of copanlisib (□), lapatinib (○) and a combination of lapatinib and copanlisib (Δ) in a panel of gastric cancer cell lines. Error bars are representative of standard deviations across triplicate experiments. The ratio of drugs is indicated on each graph. **b** Efficacy of refametinib (◊), lapatinib (○) and a combination of lapatinib and refametinib (Δ) in a panel of gastric cancer cell lines. Error bars are representative of standard deviations across triplicate experiments. The ratio of drugs is indicated on each graph. **c** The efficacy of combining trastuzumab (T) at 10 µg/ml and copanlisib (C) at varying concentrations in a panel of gastric cancer cell lines. *indicates a p-value < 0.05 as calculated by Kruskal–Wallis non-parametric test. **d** The efficacy of combining trastuzumab (T) at 10 µg/ml and refametinib (R) at varying concentrations in a panel of gastric cancer cell lines. Standard deviations are representative of independent triplicate experiments

The combination of lapatinib and copanlisib enhanced growth inhibition relative to testing either drug alone in ESO26 and OE19 cells. Lapatinib in combination with copanlisib had a synergistic response in ESO26 cells (CI@ED50 = 0.55 ± 0.19) and an additive effect in OE19 cells (CI@ED50 1.09 ± 0.45). The combination of lapatinib and copanlisib was antagonistic in NCI-N87, SNU16 and KATOIII cells (Fig. 5a).

Combinations of lapatinib and refametinib had a synergistic effect in ESO26, NCI-N87 and SNU16 cells (ESO26 CI@ED50 = 0.59 ± 0.12; NCI-N87 CI@ED50 = 0.65 ± 0.13; SNU16 CI@ED50 = 0.59 ± 0.08). The combination of lapatinib and refametinib was antagonistic in OE19 cells and the HER2-negative KATOIII cell line (Fig. 5b).

### Combinations of trastuzumab and copanlisib improve response to either drug tested alone in some HER2-positive gastric cancer cell lines

The combination of trastuzumab and copanlisib resulted in significantly improved growth inhibition compared to either therapy alone in three of the cell lines tested (ESO26, NCI-N87 and OE19) (p < 0.05) (Fig. 5c). The combination of trastuzumab and copanlisib did not enhance growth inhibition relative to either drug alone in the weakly HER2-positive SNU16 cell line or the HER2 negative KATOIII cell line. The combination of trastuzumab and refametinib did not result in enhanced growth inhibition compared to either therapy alone in any of the cell lines tested (Fig. 5d).

### Changes in the proteomic profile of HER2-positive gastric cell lines after treatment

**Fig. 6:**
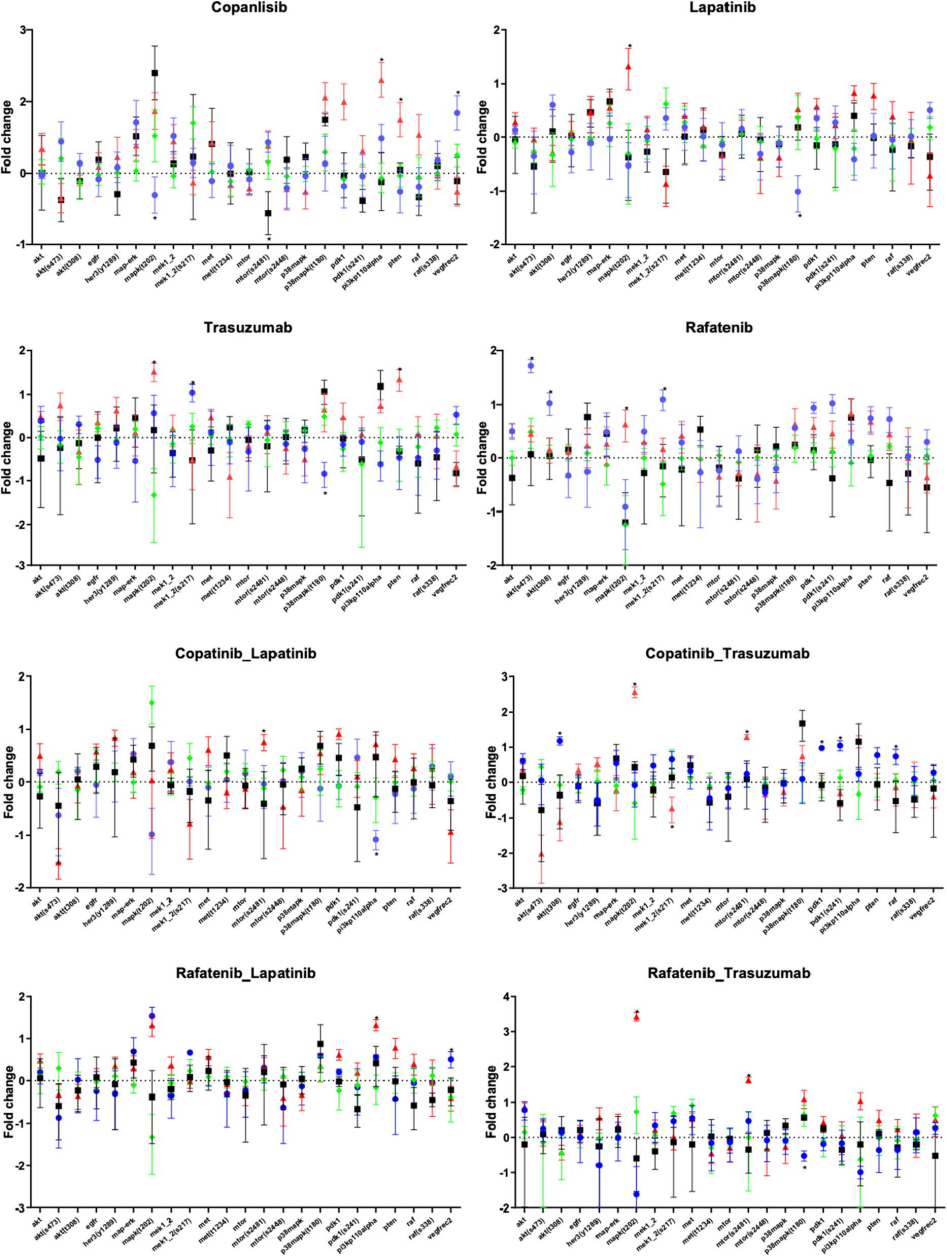
Proteomic profile of HER2-positive gastric cell lines. Protein expression was examined in SNU-16 (□), NCI-N87 (○), ESO26 (Δ), and OE19 (♦) cell lines after treatment with copanlisib, refametinib, lapatinib, trastuzumab and their combinations

Antibodies for RPPA analysis were chosen to represent multiple nodes on the PI3K/AKT and MAPK/ERK signalling pathways. Protein expression following treatment was examined in HER2-positive cell lines with different mutational status (Fig. 6). After treatment with copanlisib, expression of PDK1, PI3-Kinase p110alpha and PTEN expression were significantly higher in the ESO26 cell line compared with the other cell lines tested. There was a significant downregulation of MAPK (T202/Y204) and an upregulation VEGF Receptor 2 in the NCI-N87 cell line, which was not seen in any of the other cell lines tested. Following treatment with lapatinib, MAPK (T202/Y204) was significantly upregulated in the ESO26 cell line and p38 MAP Kinase (T180/Y182) was downregulated in the NCI-N87 cell line compared with other cell lines tested. Following trastuzumab treatment, we observed an upregulation of MEK1/2 (S217/221) and a downregulation of p38 MAP Kinase (T180/Y182) in the NCI-N87 cell line, while expression of MAPK (T202/Y204) and PTEN was upregulated in the ESO26 cell line compared with other cell lines.

Following treatment with refametenib, expression of AKT (S473), AKT (T308) and MEK1/2 (S217/221) were significantly upregulated in NCI-N87 cells. MAPK (T202/Y204) was significantly upregulated in ESO26. After co-treatment with copanlisib and lapatinib, expression of mTOR (S2481) was upregulated in ESO26. PI3-Kinase p110alpha was downregulated in NCI-N87 cells compared with the other cell lines tested. Following co-treatment with copanlisib and trastuzumab, expression of AKT (T308), PDK1, PDK1 (S241) and RAF were upregulated in NCI-N87 cells, while MAPK (T202/Y204), and mTOR (S2481) were upregulated and MEK1/2 (S217/221) was downregulated in ESO26 cells compared with the other cell lines tested. After co-treatment with refametinib and lapatinib, expression of PI3-Kinase p110alpha and VEGF Receptor 2 were upregulated in ESO26 and NCI-N87 cells, respectively, while co-treatment with refametinib and trastuzumab resulted in upregulated expression of MAPK (T202/Y204) and mTOR (S2481) in ESO26 cells. Co-treatment with refametinib and trastuzumab also resulted in decreased expression of p38 MAP Kinase (T180/Y182) in NCI-N87 cells compared with the other cell lines tested.

## Discussion

Trastuzumab is currently the only first-line targeted therapy approved for the treatment of advanced GC [8]. However, the objective response rate (ORR) of trastuzumab is lower in HER2-positive GC than in HER2-positive breast cancer (16% vs. 26%) [8, 29], and although trastuzumab treatment improves PFS and OS in HER2-positive GC, intrinsic or acquired resistance to trastuzumab is a significant clinical problem [15].

PI3K pathway activation is common in many cancers, including GC [30, 31]. While a number of studies have reported on PI3K activation as an unfavourable prognosis factor in breast cancer [18, 32, 33], the mechanisms of resistance to trastuzumab in GC are less clear [21, 34]. In this study, we sought to evaluate associations between activation of the PI3K and MAPK pathways and survival outcomes in HER2-positive and HER2-negative GCs, and investigate potential mechanisms of resistance to trastuzumab that could provide novel strategies for the treatment of HER2-positive GC.

Previous studies have suggested that continuously active HER2 downstream signalling pathways, including the PI3K and MAPK pathways, might result in the relatively lower sensitivity of trastuzumab in HER2-positive GC than in HER2-positive breast cancer [35, 36]. We have shown that 17% of HER2-positive GCs harbour somatic ERBB-family mutations, which can result in activation of the PI3K and/or MAPK pathways [37]. We have found that overall survival is significantly poorer in HER2-positive GC patients whose tumours harbour these mutations compared to HER2-negative GC patients, suggesting an unmet clinical need in the optimal treatment of this patient population. Proteomic analysis showed increased PDK1 protein expression in HER2-positive gastric tumours harbouring ERBB-family mutations compared to wildtype tumours. PDK1 is required for the activation of AKT, and increased PDK1 expression in breast cancer is associated with ERBB2 amplification and PIK3CA mutations. Additionally, increased PDK1 potentiates AKT signalling in the setting of upstream PI3K pathway activation [38]. PDK1 has been shown to activate the MAPK signalling pathway by directly phosphorylating MAK1/2 [39]. Therefore, targeting the PI3K or MAPK signalling pathways could potentially represent an improved treatment strategy and help to improve trastuzumab sensitivity, particularly in tumours harbouring PIK3CA and/or ERBB-family mutations.

Several studies have investigated targeting the PI3K pathway in GC. The dual PI3K/mTOR inhibitor BEZ235 demonstrates anti-tumour activity in vitro and in vivo in GC models [35, 40]. However in the clinical setting, results with BEZ235 have been disappointing, with toxicity and a lack of clinical efficacy reported [41]. Similarly, the mTOR inhibitor everolimus did not demonstrate a survival benefit in patients with advanced GC in the GRANITE-1 study [42], likely in part because inhibition of mTOR activates a feedback loop which upregulates PI3K activity, thereby attenuating the anti-tumour efficacy of mTOR inhibitors [43]. However, targeting PI3K directly is likely to represent a more optimal treatment strategy. Pan-isoform PI3K inhibitors, which target the p110 subunit of PI3K, are generally well tolerated and several of these agents are currently in phase II or III clinical trials. Isoform specific inhibitors have more focused toxicities that allow them to be used at higher doses, resulting in more complete and reliable inhibition of PI3K.

In this study we have used copanlisib, a selective alpha/delta isoform dominant PI3K inhibitor with in vivo and in vitro activity [44, 45]. We have previously shown that copanlisib can restore sensitivity to trastuzumab and lapatinib in vitro in HER2-positive breast cancer cell lines with acquired resistance to trastuzumab and/or lapatinib [24]. We have also found the combination of copanlisib and trastuzumab to be safe and well tolerated in a phase Ib clinical trial in patients with recurrent or metastatic HER2-positive breast cancer who are resistant to trastuzumab [46]. In the current study, combining copanlisib with lapatinib resulted in greater proliferation inhibition relative to testing either drug alone in three out of four HER2-positive cell lines tested. The NCI-N87 cell line, where the combination of lapatinib and copanlisib was not additive or synergistic, contains two mutations in the ERBB2 gene. Previous studies have shown that mutations in ERBB2 may increase HER3 phosphorylation [47], which can attenuate the anti-tumour effect of some PI3K inhibitors [48]. However, this is unlikely to be the reason in our study as NCI-N87 cells were particularly sensitive to both single agent lapatinib and single agent copanlisib lapatinib IC_50_: 39.8 ± 13.3 nM; copanlisib IC_50_: 26.8 ± 9.7 nM). When we combined trastuzumab with copanlisib, we again observed greater proliferation inhibition relative to either drug alone in two out of the four HER2-positive cell lines tested. The only cell line where we did not observe this was the SNU16 cell line, which is only very weakly HER2-positive. Interestingly, the combination of trastuzumab and copanlisib was effective in the OE19 cell line, which was resistant to either drug as a single agent.

The role of MEK inhibition in the treatment of HER2-positive GC is less clear. Refametinib is a potent allosteric MEK1/2 inhibitor that we, and others, have shown to have anti-proliferative effects, including in HER2-positive breast cancer cells [25, 49]. We have shown that refametinib, like copanlisib, can restore sensitivity to cells with acquired resistance to lapatinib [25]. Refametinib, used either alone or in combination with targeted therapies, was well tolerated in phase I and phase II clinical studies in patients with advanced solid tumours and demonstrated some clinical benefit [50–52]. In our study, the combination of lapatinib and refametinib was synergistic in all HER2-positive cell lines tested, apart from the OE19 cell line. However, of the cell lines that we tested OE19 cells were the most sensitive to single agent refametinib (IC_50_: 2.75 ± 0.37 µM). The combination of refametinib and trastuzumab did not result in greater proliferation inhibition relative to testing either drug alone in any of the cell lines we tested. However, the HER2-positive OE19 and SNU16 cell lines, as well as the HER2-negative KATOIII cell line were sensitive to single agent refametinib, with IC_50_s ranging from 2.75 ± 0.37 µM in OE19 cells to 6.05 ± 0.84 µM in KATOIII cells.

In summary, ERBB-family mutations in HER2-positive GC may result in increased signalling through the PI3K and MAPK signalling pathways. PI3K and MAPK may therefore represent a rational strategy to improve responses to anti-HER2 therapy in GC. In vitro we have shown that the PI3K inhibitor copanlisib and the MEK inhibitor refametinib have anti-proliferative effects as monotherapy in some HER2-positive GC cell lines, including those that are intrinsically resistant to trastuzumab. Interestingly, combinations of copanlisib or refametinib and lapatinib also induce additive or synergistic anti-proliferative effects in some cell lines. The combination of copanlisib and trastuzumab also offers greater benefit than using either drug alone. Our data suggest that the addition of copanlisib to HER2-targeted therapy should be considered for clinical trial evaluation in patients with HER2-positive GC, particularly in those patients with PI3K pathway activated tumours.

## Supplementary Information

**Additional file 1: Table S1. **Nonsynonymous somatic mutations in PIK3CA and ERBB family genes 108 Non-synonymous mutations in EGFR, ERBB2, ERBB3, ERBB4 and PIK3CA analysed by Agena MassArray in this study. **Table S2.** The full list of antibodies used in the RPPA assay.

**Additional file 2: Table S3.** Raw data from RPPA experiments.

## Data Availability

The datasets analysed during the current study are available from the corresponding author on reasonable request.
