# Supplementary material for: Targeting the PI3K and MAPK pathways to improve response to HER2-targeted therapies in HER2-positive gastric cancer
Source: J Transl Med. 2021 May 1;19:184. doi: 10.1186/s12967-021-02842-1 (PMC8088633; doi:10.1186/s12967-021-02842-1)
Supplement: Supplementary file 1 — Additional file 1: Table S1. Nonsynonymous somatic mutations in PIK3CA and ERBB family genes 108 Non-synonymous mutations in EGFR, ERBB2, ERBB3, ERBB4 and PIK3CA analysed by Agena MassArray in this study. Table S2. The full list of antibodies used in the RPPA assay. [file 12967_2021_2842_MOESM1_ESM.docx]

**SUPPLEMENTARY TABLES**

| **EGFR** | **ERBB2** | **ERBB3** | **ERBB4** | **PIK3CA** |
| --- | --- | --- | --- | --- |
| G179A/D | S310F/Y | V104M | S303Y/F | R88Q |
| V689M | L755S | A232V | V348M/L | K111N |
| N700D | D769H | P262H | D595G/V | N345K |
| E709A/V/G/K/Q | G776S/V | G284R | V721I | C420R |
| G719C/S/R | V777L/M/A | D297Y | R782Q | E453K |
| S720T/P | V842I | T355A | E810K | E542V/G/K/Q |
| D761N/Y | H878Y | G325R | P854Q | E545K/Q/D/A/G/V |
| V769L/M |  | T389K | N861Y | Q546H/L/P/R/E/K |
| T783A |  | V714M | E872K/V | Y1021H/N/C |
| A839T |  | Q809R | E874X | R1023Q |
| K846R |  | S846I | T926M | T1025I/A/S |
| L858M |  | E928G | E934K | A1035V/T |
| L858R |  |  | K935T/R/I | M1043V/I |
| L861Q/R  G863D  H870R |  |  | K935E  G936R | A1046V  H1047R/L/Y  G1049R |
|  |  |  |  |  |

Table S1: Nonsynonymous somatic mutations in PIK3CA and ERBB family genes 108 Non-synonymous mutations in EGFR, ERBB2, ERBB3, ERBB4 and PIK3CA analysed by Agena MassArray in this study

| **Antibody** | **Cat#** | **Company** | **Dil** | **Host** |
| --- | --- | --- | --- | --- |
| Akt | 4691 | CellSig | 1:3000 | R |
| AKT (S473) | 9271 | CellSig | 1:250 | R |
| AKT (T308) | 2965 | CellSig | 1:500 | R |
| Akt2 | 2964 | CellSig | 1:50 | R |
| MAPK - ERK 1/2 | 9102 | CellSig | 1:200 | R |
| MAPK (T202/Y204) -ERK1/2 | 4377 | CellSig | 1:1200 | R |
| MEK1 | 1235-1 | Epitomics | 1:1200 | R |
| MEK1/2 (S217/221) | 9154 | CellSig | 1:1000 | R |
| mTOR | 2983 | CellSig | 1:400 | R |
| mTOR (S2448) | 2971 | CellSig | 1:100 | R |
| p38 MAP Kinase (T180/Y182) | 9211 | CellSig | 1:250 | R |
| p38_MAPK | 9212 | CellSig | 1:300 | R |
| p70 S6 Kinase | 1494-1 | Epitomics | 1:250 | R |
| p70 S6 Kinase (T389) | 9205 | CellSig | 1:250 | R |
| PDK1 | 3062 | CellSig | 1:100 | R |
| PDK1 (S241) | 3061 | CellSig | 1:500 | R |
| PI3-Kinase p110alpha | 4255 | CellSig | 1:100 | R |
| PKC-alpha | 05-154 | Upstate | 1:2000 | M |
| PKC-alpha (S657) | 06-822 | Upstate | 1:3000 | R |
| PTEN | 9552 | CellSig | 1:1000 | R |

Table S2: The full list of antibodies used in the RPPA assay

AKT, protein kinase B; MAPK, mitogen activated protein kinase; MEK1, mitogen activated protein kinase kinase; mTOR, mammalian target of rapamycin; p70 S6 kinase, ribosomal protein S6 kinase beta-1; PDK1, phosphatidylinositide-dependent protein kinase 1; PKC, protein kinase C; PTEN, Phosphatase and tensin homolog. CellSig, Cell Signaling Technology, Netherlands; Epitomics, An Abcam Company, USA; Upstate, Merck Millipore USA.
